# Thompson's quadricepsplasty for stiff knee

**DOI:** 10.4103/0019-5413.37004

**Published:** 2007

**Authors:** ZS Kundu, SS Sangwan, G Guliani, RC Siwach, P Kamboj, Raj Singh

**Affiliations:** Department of Orthopaedics Pt. B.D. Sharma PGIMS, Rohtak (Haryana), India

**Keywords:** Knee stiffness, Thompson's quadricepsplasty, continuous passive motion

## Abstract

**Background:**

Stiffness of the knee after trauma and/or surgery for femoral fractures is one of the most common complications and is difficult to treat. Stiffness in extension is more common and can be reduced by vigorous physiotherapy. If it does not improve then quadricepsplasty is indicated. The present study was undertaken to evaluate the results of Thompsons quadricepsplasty.

**Materials and Methods:**

Twenty-two male patients (age range 20-45 years) with posttraumatic knee stiffness following distal femoral fractures underwent Thompson's quadricepsplasty where knee flexion range was less than 45°. The index injury in these patients was treated with plaster cast (n=5), plates (n=3), intramedullary nailing (n=3) and external fixator for open fractures (n=9). Thompson's quadricepsplasty was performed in all the patients using anterior approach, with incision extending from the upper thigh to the tibial tubercle. Release of rectus femoris from underlying vastus intermedius and release of intraarticular adhesions were performed. After surgery the patients needed parenteral analgesia for three days and then oral analgesics for three weeks. Active assisted knee mobilization exercises was started on the first postoperative day. Continuous passive motion machine was used from the same day. Supervised physiotherapy was continued in hospital for six weeks followed by intensive knee flexion and extension exercise including cycling at home for atleast another six months.

**Results:**

Out of 22 patients, 20 had excellent to good results and two patients had poor results using criteria devised by Judet. One poor result was due to peroperative fracture of patella which was then internally fixed and hence the flexion of knee could not be started immediately. There was peroperative avulsion of tibial tuberosity in another patient who finally gained less than 50° knee flexion and hence a poor result.

**Conclusion:**

Thompsons quadricepsplasty followed by a strict and rigourous postoperative physiotherapy protocol successfully increases the range of knee flexion.

External fixators with or without crossing the knee joint are commonly used in open fractures of distal femur.^1^ In such cases chances of knee stiffness are high. Knee stiffness is one of the most common complications following intramedullary or extramedullary fixation for distal femoral fractures. Knee flexion less than 45° causes problems in gait and hindrance in day to day activities. Some degree of knee movements can be increased by gentle manipulation under anesthesia but there are chances of hemarthrosis and recurrence of stiffness. Intensive physiotherapy may gain movements sufficient for routine activities only in a few cases. Quadricepsplasty is the surgical procedure required to release the quadriceps muscle in order to improve the range of knee flexion. This procedure is indicated mainly for stiffness in extension.^2^–^4^ Thompson and Judet type of quadricepsplasties are the most common surgical procedures described to treat knee stiffness, the former being more popular.^5^ We evaluated our results after Thompson's quadricepsplasty and physiotherapy.

## MATERIALS AND METHODS

Twenty-two male patients with posttraumatic knee stiffness with age ranging from 20 to 45 years, underwent Thompson's quadricepsplasty for severe extension contractures between March 1999 to June 2004. Fracture of the distal femur was the original injury in all these patients. All patients had less than 45° range of knee flexion. Nine patients with open fractures were treated with external fixator; eight patients developed postoperative stiffness following internal fixation in severely comminuted distal femoral fractures whereas five treated nonoperatively with plaster cast, developed residual knee stiffness. All these patients had original injuries in road-traffic accidents. The preoperative range of knee flexion ranged from 5° to 45° (average 21°). The patients were operated after at least one and a half years of original injury. A preoperative assessment as to the site of probable adhesions was made both clinically and radiologically. A note was made of side to side patellar movements, passive knee range of motion and any tightness of the rectus femoris over a healed scar due to injury, surgery or an adjoining pin tract site. Radiologically, any fracture callus, beak over the anterior surface of the distal femur or articular incongruity was adjudged to be a site of probable adhesion. All the patients were followed up for a period of more than two years (range two to five years).

### Surgical procedure:

**Figure 1:**
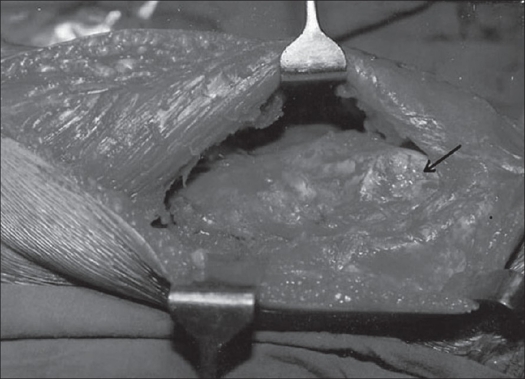
Rectus femoris isolated from fibrosed vastus intermedius, arrow showing femoral condyle

**Figure 2:**
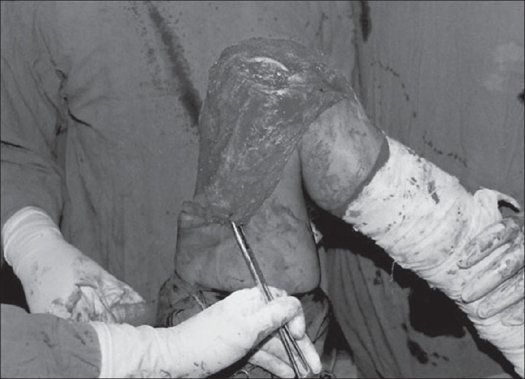
Peroperative flexion achieved

Patients were operated either under general (n = 2) or spinal anesthesia (n = 20) in supine position without tourniquet. A midline incision extending from the upper fourth of the anterior aspect of the thigh up to the tibial tuberosity was given. Main dissection was centered at the site of adhesions adjudged clinicoradiologically preoperatively and those encountered peroperatively. Extensor expansions of the knee were released on both sides of the patella and intra-articular fibrotic band-like adhesions, found in all cases, were released using a sharp dissection. In all the cases there were extra-articular as well as intra-articular adhesions. Rectus femoris was separated from vastus intermedius [Figure 1]. The vastus intermedius was released and excised extraperiosteally in all cases. Any projecting bony spike anteriorly was also removed by nibbling and raw bone was covered with fat graft (n=1). Vastus lateralis and vastus medialis were released at least up to the upper third of the thigh. The entire procedure was performed with sharp dissection using electric cautery for achieving hemostasis. Following excision of vastus intermedius and after release of the rest of quadriceps, gentle manipulation was performed to achieve at least more than 90° knee flexion. In 19 patients we were able to achieve preoperatively more than 100° knee flexion [Figure 2]. Meticulous hemostasis was achieved and negative suction drains were inserted to prevent hematoma formation. Only a few loose stitches were applied to appose the retinaculum on the sides of the patella, while keeping the knee just short of 90° flexion. Only skin closure was done. After skin closure, range of knee movements were about 15° less than what was achieved before closure.

### Postoperative regimen and followup:

In the immediate postoperative period a well-padded crepe bandage was applied, limbs were raised on Braun-Bohler's splint and pillows and ice packs were applied for 72h. Parentral analgesics were given on the first three postoperative days and further oral analgesics were continued for another three weeks. CPM was started on first postoperative day with 0° to 60° flexion in the first week. In between, patients were advised active assisted quadriceps exercises, particularly extension of the knee. Patients were taught all these exercises preoperatively so that they could perform these exercises better in the postoperative period even in spite of pain. The CPM range was gradually increased until maximum possible flexion could be achieved within two weeks time. Patients were discharged after achieving at least 90° flexion and usually two to three days after the stitch removal. After discharge from the hospital all these patients were kept under supervised institutional physiotherapy for another six weeks on regular day to day outpatient basis. Additional flexion and quadriceps strengthening exercises, isometric quadriceps exercises, resistance exercises, hamstring exercises including cycling were encouraged at homes for another six months.Patients were kept non weight bearing for two weeks till stitch removal. They were followed every third week for the first three months and subsequently at six-monthly intervals for at least two years. The range of followup was two to five years. The results were assessed according to Judet's criteria and were considered excellent if final flexion was greater than 100°; good if flexion was between 80° to 100°; fair if flexion was between 50° and 80° and poor, if flexion was less than 50°.

## RESULTS

Patients profile, complications and results are discussed in detail in Table 1. Average range of preoperative flexion was 21° (range 5° to 45°). The range of intraoperative flexion was 90° to 120° (average 102° flexion) in all except one, where peroperative fracture patella occured. None of the patients had V-Y plasty of rectus femoris and we avoided it even in the cases where we could not achieve more than 90° peroperative flexion as it is a major cause of disabling extension lag. After a followup of at least two years, the range of flexion was 85° to 120° [Figure 3a and b] with mean of 98° in all except in two poor results. The average loss of flexion in the postoperative period was 11° of what was achieved intraoperatively. Two patients had problems peroperatively. One had fracture of lower pole of patella and other had undisplaced avulsion of tibial tuberosity. In the first patient internal fixation using cerclage wiring was done and another was treated conservatively. These two patients had poor results with ultimate gain in flexion of 50° each. The mean hospital stay was 17 days ranging from 14 to 21 days.

**Table 1:** Observations and results

Pt. No.	Age (Yrs)	Primary T/t following fracture femur	Preoperative Flexion (deg.)	Intra-op Flexion (deg.)	Flexion Loss (deg.)	Final Flexion (deg.)	Flexion Gain (deg.)	Complications	Ext. Lag	Results
1	22	IM nail	10	120	0	120	110	-	5	Exce.
2	20	POP cast	30	110	10	100	70	-	-	Good
3	44	Ex. Fix.	25	100	5	95	70	-	-	Good
4	45	Ex. Fix.	15	95	5	90	75	-	-	Good
5	32	Ex. Fix.	15	90	0	90	75	-	-	Good
6	43	POP cast	10	100	50	50	50	Avuls. Ti. Tub.	-	Poor
7	21	Ex. Fix.	10	110	10	100	90	-	5	Good
8	29	POP cast	10	115	15	100	90	-	-	Good
9	32	DCS	15	120	5	115	100	-	-	Exce.
10	22	Ex. Fix.	35	100	5	95	65	-	-	Good
11	42	DCS	5	50	50	50	Nil	# Patella	-	Poor
12	36	Plating	40	120	10	110	70	-	-	Exce.
13	38	DCS	40	100	10	110	70	-	-	Exce.
14	40	Ex. Fix.	30	90	5	85	55	-	5	Fair
15	25	POP cast	15	105	10	95	80	-	-	Good
16	29	POP cast	10	95	5	90	80	Hemat.	-	Good
17	31	Ex. Fix.	20	100	5	95	75	-	-	Good
18	37	Ex. Fix.	25	100	5	95	70	-	-	Good
19	24	Ex. Fix.	10	100	10	90	80	-	-	Good
20	27	IM Nail	15	95	5	90	75	-	7	Fair
21	22	IM Nail	30	105	10	95	60	-	-	Good
22	21	DCS	45	120	20	100	55		-	Good

Pt.- Patient, T/t - Treatment, Preoperative - Preoperative, Intra-op - Intraoperative, Deg. - Degrees, IM - Intramedullary, POP - Plaster of Paris, Ex. Fix. - External Fixator, DCS - Dynamic Condylar Screw, Avuls. Ti. Tub. - Avulsion Tibial Tuberosity, Exce. - Excellent, Hemat. - Hematoma, Ext. - Extension, # - Fracture

**Figure 3a:**
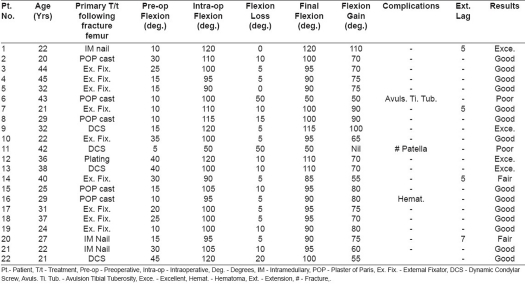
Preoperative flexion 15°

**Figure 3b:**
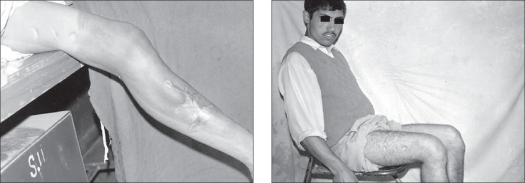
Postoperative flexion 95°

Using these criteria there were four excellent, 14 good, two fair and two poor results due to the peroperative complications.

## DISCUSSION

Quadricepsplasty is the recommended procedure for release of severe knee extension contracture. The post traumatic knee stiffness can impose a severe handicap and disability that can threaten the occupational and leisure activities of the patient. Knee flexion of less than 70° hampers the normal gait of the patient and produces limp.^5^ The pathological alterations that cause a block to knee flexion are fibrosis and shortening of the medial and lateral parapatellar retinaculum, adhesions between the deep surface of the patella and femoral condyle, fibrosis of the vastus intermedius with adherence to the rectus femoris muscle and to the front of femur; and actual shortening of the rectus femoris.^4^,^6^,^7^ Additionally fracture callus and adhesion of the skin to underlying muscles particularly in open fractures treated with external fixators at pin and scar site should also be considered in the causes of limitation of flexion.^5^

Thompson described this procedure which is based principally on isolating the rectus femoris completely from the vasti and releasing it in such a manner that it takes over the action of knee extension.^2^ If the rectus femoris is quite tight it needs V-Y plasty and lengthening which can lead to extensor lag which is a disabling problem. This problem has been widely reported in the literature.^4^,^7^,–^17^ In our series, even in the cases where there was 90° peroperative flexion we did not perform V-Y plasty of rectus femoris. Hence in all cases we relied on the adequate release of scarred and adherent tissues and on separation of rectus femoris from underlying vastus intermedius. By extending the dissection and release more proximally, flexion of 90° or more could be achieved.

Paley, however, advocated Judet's quadricepsplasty for extension knee contractures stating that it permits a controlled, sequential release of the intrinsic and then the extrinsic components limiting knee flexion. At any phase of the procedure, if adequate flexion is obtained, the dissection is stopped. This greatly reduces the potential for iatrogenic quadriceps rupture or extensor lag and limits the potential dissection.^18^

We educated our patients on the role of active physiotherapy after surgery. For achieving good amount of intraoperative flexion meticulous extraperiosteal dissection and excision of vastus intermedius from bone and the separation of rectus femoris is essential. Dissection and release should extend upto the upper third of the thigh. We observed intraarticular as well as extraarticular adhesions in all cases. Therefore release should be aimed at both sites. Any protruding bony fragment on the anterior aspect of distal femur was excised and the raw bone was covered with fat graft and stitched to neighboring soft tissues in order to allow proper excursion of rectus femoris muscle.

The surgical procedure encompasses meticulous hemostasis, use of cautery for dissection, negative suction drain, use of ice-packs and elevation of limb postoperatively to decrease the chances of edema and swelling, thus minimizing the formation of adhesions and loss of achieved flexion.^5^,^19^ To prevent extensor lag V-Y plasty should be avoided and vigorous postoperative active quadriceps extension exercises are strictly emphasized. The patient should be explained preoperatively that the gain of knee range of flexion may be at the cost of a persistent mild extensor lag.^1^,^4^,^14^ The importance of education of knee quadriceps as well as hamstring exercises in preoperative period should be taught to the patient and emphasized adequately that these will later be required in the immediate postoperative period. These exercises will prevent the quadriceps inhibition in the painful postoperative period. Continuous passive motion and vigorous postoperative active knee exercises reduce the formation of adhesions, prevent quadriceps inhibition,allow gain in knee flexion and decrease the chances of extensor lag.

Judet *et al.*^20^ reported 53 cases with 85% (45 patients) good to excellent results. Nicoll^4^ reported only 33% (10) patients had good or excellent results. Hesketh^8^ reported results of Thompsons quadricepsplasty in ten cases. The average gain in flexion was 95°. All his patients attained over 100° of knee flexion except one. Daoud *et al*.^15^ 1982 reported results of six patients operated by Judet's technique. The average range of flexion was 0-115°. Sprague NF *et al*.^21^ lysed knee arthrofibrosis in 24 patients by arthroscopic methods and achieved over 100° flexion in 18 patients. Ali *et al*.^5^ (2003) in their series of 10 patients treated by Judets technique reported nine good or excellent and one fair result. Wang *et al*.^19^ operated 22 patients by their innovative technique and achieved excellent results in 16 knees, good in five and fair in one. We have achieved excellent to good results in 20 out of 22 cases.

The quadricepsplasty is a major operation; its ability to successfully achieve knee flexion range depends both on the surgeon as well as the patient. To achieve a successful outcome,preparation begins in the preoperative period with patient education on knee exercises; continues in the peroperative period by use of meticulous sharp dissection and hemostasis, and is followed up postoperatively by adhering to a strict postoperative rigourous physiotherapy protocol in conjuction with sound will and the ability of the patient so as to prevent quadriceps inhibition and allow toning and strengthening of the quadriceps and hamsrings muscles. The patient must be sensitized about mild extensor lag which he may have on followup.
